# GEISHA: an evolving gene expression resource for the chicken embryo

**DOI:** 10.1093/nar/gkt962

**Published:** 2013-10-21

**Authors:** Parker B. Antin, Tatiana A. Yatskievych, Sean Davey, Diana K. Darnell

**Affiliations:** Molecular Cardiovascular Research Program, Department of Cellular and Molecular Medicine, University of Arizona, Tucson, AZ 85724, USA

## Abstract

GEISHA (Gallus Expression *In Situ* Hybridization Analysis; http://geisha.arizona.edu) is an *in situ* hybridization gene expression and genomic resource for the chicken embryo. This update describes modifications that enhance its utility to users. During the past 5 years, GEISHA has undertaken a significant restructuring to more closely conform to the data organization and formatting of Model Organism Databases in other species. This has involved migrating from an entry-centric format to one that is gene-centered. Database restructuring has enabled the inclusion of data pertaining to chicken genes and proteins and their orthologs in other species. This new information is presented through an updated user interface. *In situ* hybridization data in mouse, frog, zebrafish and fruitfly are integrated with chicken genomic and expression information. A resource has also been developed that integrates the GEISHA interface information with the Online Mendelian Inheritance in Man human disease gene database. Finally, the Chicken Gene Nomenclature Committee database and the GEISHA database have been integrated so that they draw from the same data resources.

## INTRODUCTION

A central tenet of modern biomedical research is that understanding gene function will lead to improvements in human health. Basic and applied research into mechanisms of evolutionary, molecular, cellular and developmental biology and agriculture are dependent on the linkage between the genome and its biological output. Crucial information for all of these disciplines is to know when and where genes are expressed. In the context of embryonic development, the precise temporal and spatial gene expression information provided by mRNA *in situ* hybridization (ISH) lends immediate insight into potential function.

Of the dozen or so model organisms most relevant to biomedical research, several attributes of the chicken embryo make it particularly amenable to large-scale ISH expression screening (1). These include advantageous morphology, unlimited availability of embryos at low cost and embryo morphology that is highly similar to human. As chicken is also the first bird species to have its genome sequenced (2), it often serves as a reference species for avian research and genomics.

As the quantity of ISH expression data rapidly increased in the 1990s, several databases were developed to house and display ISH images and metadata. For organisms having a recognized Model Organism Database (MOD), this information was often incorporated into the MOD for the respective species (3–5). A MOD for chicken had not been developed, and so the GEISHA (Gallus Expression *In Situ* Hybridization Analysis) project was launched to acquire, store and display ISH gene expression information and associated metadata for the chicken embryo (6). This update describes important enhancements to GEISHA since our last report (7). Improvements include restructuring the database and user interface from an entry- to a gene-centric format concordant with MODs in other organisms. This reorganization has enhanced the integration within the database and user interface of genomic, transcript, protein and expression data between chicken and other model organisms. Enhanced search and browse features have been added, and a human disease gene resource now integrates expression information between GEISHA entries and gene mutations linked to human disease. Finally, the Chicken Gene Nomenclature Committee (CGNC) database has been integrated with GEISHA to unify resource information for chicken genes, gene expression and other genomic data.

## GEISHA: A GATEWAY FOR CHICKEN GENE AND GENE EXPRESSION INFORMATION

### Structural reorganization

The GEISHA project was initiated in the late 1990s before sequencing of the chicken genome. Lacking gene models, ISH images and metadata were conceptually organized around data entries (6). Availability of the chicken genome assembly and the genomes of dozens of other vertebrate and invertebrate organisms provided a framework for integrating expression and genomic information in chicken and other species. Accordingly, the underlying GEISHA database was restructured from one organized around ISH entries and images to one centered on genes. Support for integration with similar data from other model species was also added. The gene data support code was rewritten from an original code based on Ensembl data to one based on NCBI Entrez Gene data. These large changes required a retooling of the search infrastructure. The user interface presentation layer was rewritten to work with the most recent HTML/CSS standards and practices.

**Figure 1.**
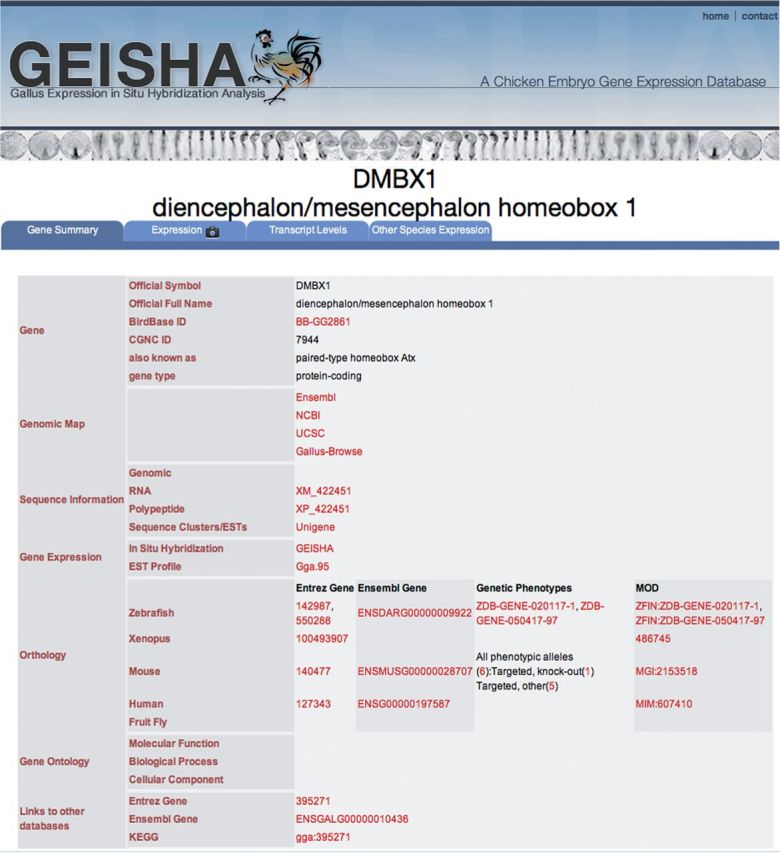
Sample gene summary page, in this case for the gene *DMBX1.* Data are accessed through four tabs: ‘Genes Summary’ (shown); ‘Expression’, showing chicken ISH images and anatomical location of expression; ‘Transcript Levels’, showing quantitative mRNA expression data; and ‘Other Species Expression’, showing links to ISH expression information in fruitfly, frog, zebrafish and mouse.

**Figure 2.**
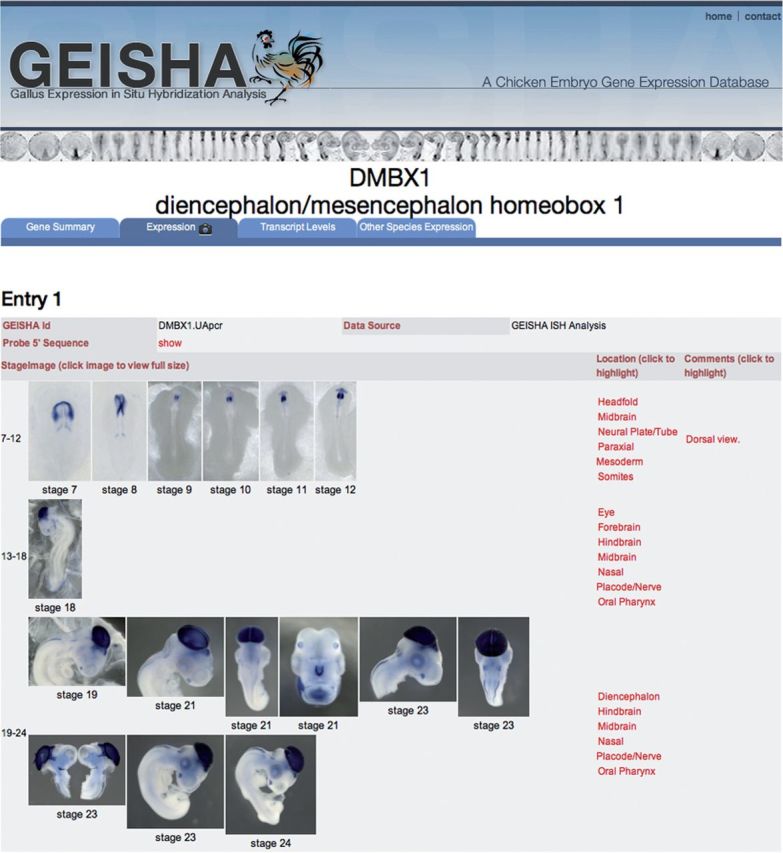
Expression summary page for the gene *DMBX1*. ISH data are organized into entries according to the source date. ISH data shown in Entry 1 were generated from an in house ISH screen (GEISHA Id: DMBX1.UApcr). Additional entries (not shown) present expression data using other probes or curated from publications. Images are organized by embryo stage. Anatomical location of expression is shown to the right of the images. Clicking on a thumbnail opens a high-resolution image.

This reorganization provided the framework to incorporate a large amount of information pertaining to chicken genes and proteins and their orthologs in other species. The user interface has similarly been reorganized to logically present different types of information to users (Figure 1). A ‘Gene Summary’ tab provides (i) gene, transcript and protein information; (ii) links to external information sources that include genomic and gene expression resources; (iii) information about orthologous genes in zebrafish, frog, mouse, fly and human; (iv) links to corresponding model organism data pages; and (v) links to any ortholog in the human Online Mendelian Inheritance in Man (OMIM) resource. An ‘Expression’ tab shows ISH entries for the corresponding chicken gene (Figure 2), whereas the ‘Transcript Levels’ tab reveals graphical representations of mRNA transcript-level data obtained from the NCBI Unigene Database. An ‘Other Species Expression’ tab provides direct access to ISH expression information in other model organisms. Thus, GEISHA allows users to do immediate comparisons across model species for both normal expression and human disease analysis.

### Data acquisition

ISH images and metadata are principally acquired via high-throughput ISH screens and curation of the published literature. A modest number of high-quality unpublished ISH images have also been obtained from other research laboratories. Since our last report (7), ISH screens have focused on gene families. Gene family information for chicken orthologs is obtained from the HUGO Gene Nomenclature Committee (HGNC), which has grouped approximately half of all human genes into >250 gene families (8,9). Because gene families are defined as groups of genes descended by duplication from a common ancestral gene, family members often show conserved functions. A comprehensive comparison of family member expression provides a foundation for research into protein function, evolution and developmental biology. The nuances of effective biomedical intervention are also often dependent on targeting specific differences between family members. Thus, the new gene family emphasis in GEISHA enriches the many avenues of research that build from this foundation.

A new literature curation tool has also been added, automating acquisition of publications containing relevant chicken ISH information. Manuscripts are mined for images and associated metadata (gene names, probe sequence, expressing anatomy, stage, etc.).

### Querying and browsing GEISHA

The GEISHA database serves a broad spectrum of biologists with interests that include embryo development, biomedicine, evolutionary biology and poultry science. With this diversity of users, we have endeavored to offer easy and intuitive data access while maintaining sophisticated search and query capabilities. The GEISHA database presently contains >36 000 images of whole-embryo ISH and embryo sections from embryonic days 0–5. Some data from older embryos focus on late developing tissues (e.g. feathers). Metadata associated with all images can be browsed and searched using a variety of criteria, including embryonic stage, structural anatomical localization, gene name or ID, Gene Ontology (GO) terms and in cases where data were acquired from a publication, any author’s name. A multiparameter search incorporates any combination of these criteria. With increasing coverage of chicken proteins by GO terms, a gene family quick search has been developed that uses the most frequent GO terms associated with database entries, including transcription factors, growth factors, receptors and microRNAs.

Another enhancement is the incorporation of OMIM human disease gene information into the GEISHA database and user interface. A search interface adapted from an interface created by the GUDMAP project (10) permits users to search or browse by human disease or gene and retrieve gene and expression information for chicken orthologs.

### Integrating the CGNC and GEISHA databases

CGNC is the recognized international resource for standardized chicken gene nomenclature (11). The CGNC assigns unique gene symbols and gene names to chicken gene models according to guidelines established by the avian research community. CGNC works in conjunction with Ensembl and NCBI and draws gene name and orthology information from the HGNC (8). Where human:chicken ortholog pairs have been identified, the human gene nomenclature is used. For genes lacking obvious 1:1 human orthologs, the CGNC arrives at gene names based on input from researchers and the best available orthology information. NCBI regularly draws gene nomenclature information from CGNC to update their chicken gene information. For the last several years, CGNC has maintained a stand-alone database. With restructuring of the GEISHA database, the CNGC and GEISHA databases have been integrated to draw on the same data resources. CGNC is accessible at http://www.birdgenenames.org.

## CONCLUSION AND FUTURE PLANS

The GEISHA database is an expanding repository of chicken ISH images and associated metadata. ISH data are obtained from our high-throughput screens focused on differentially expressed genes and gene families, high-quality image collections from other laboratories and partially automated curation from the published literature. Recent improvements include a gene-centric organization, enhanced integration of chicken genomic, transcript, protein and expression and data from MODs, NCBI and Ensembl, coordinated searches with OMIM, integration of CGNC and GEISHA and a more modern and functional user interface. Future goals include completing high-throughput screening of all differentially expressed genes in the early chick embryo, adding serial section images and search capabilities and maintaining a comprehensive harvesting of published chicken ISH images/metadata. We are also focused on continued integration with other online resources, including the eChickAtlas database of 3D expression information (12).

## FUNDING

National Institutes of Health, National Child Health and Human Development Institute [P41HD064559 to P.B.A.]. Funding for open access charge: NIH [P41HD064559].

*Conflict of interest statement*. None declared.
